# Deep Learning–Based Reconstruction in Ultra-Low-Dose CT of the Ankle and Foot: A Comparative Study with Hybrid Iterative Reconstruction

**DOI:** 10.3390/tomography12100138

**Published:** 2026-09-22

**Authors:** Chuluunbaatar Otgonbaatar, Jin Woo Kim, Sung-Jin Cha, Sang-Hyun Jeon, Gonchigsuren Dagvasumberel, Hackjoon Shim, Young Hwan Jang, Jhii-Hyun Ahn, Sung Min Ko, Hyunjung Kim

**Affiliations:** 1 Medical Imaging AI Research Center, Canon Medical Systems Korea, Seoul 06173, Republic of Korea; chuka_area17@yahoo.com (C.O.); hjshim@yuhs.ac (H.S.); 2 Department of Radiology, School of Medicine, Mongolian National University of Medical Sciences, Ulaanbaatar 14210, Mongolia; mongolia.radiology@gmail.com; 3 Department of Radiology, Wonju Severance Christian Hospital, Wonju College of Medicine, Yonsei University of Korea, Wonju 26426, Republic of Korea; sunny-cocktail@hanmail.net (J.W.K.); cktjdwls9612@naver.com (S.-J.C.); leadledled@naver.com (S.-H.J.); radajh@yonsei.ac.kr (J.-H.A.); ksm9723@yahoo.co.kr (S.M.K.); 4 ConnectAI Research Center, Yonsei University College of Medicine, Seoul 03722, Republic of Korea; 5 Department of Orthopedic Surgery, Wonju Severance Christian Hospital, Wonju College of Medicine, Yonsei University of Korea, Wonju 26426, Republic of Korea; jyh3330@naver.com

**Keywords:** bone fracture, computed tomography, image reconstruction, ankle, foot, deep learning

## Abstract

**Objectives:** To evaluate the quantitative and qualitative performance of ultra-low-dose CT with deep learning image reconstruction (DLR) compared to conventional hybrid iterative reconstruction (IR) in patients with ankle and foot fractures. **Methods:** A total of 32 patients (mean age, 54 ± 16 years) with ankle and foot fractures were included in this retrospective study. All patients underwent ultra-low-dose CT imaging (effective dose, 0.86 ± 0.11 μSv), and the same CT raw data were reconstructed using both DLR and hybrid IR. Image noise, signal-to-noise ratio (SNR), and contrast-to-noise ratio (CNR) were quantitatively measured, and image sharpness was evaluated using a no-reference perceptual sharpness metric. Subjective image quality was assessed by a board-certified radiologist and an orthopedic surgeon using a five-point scale. **Results:** DLR significantly reduced image noise (47.33 ± 6.60 HU) compared to hybrid IR (87.65 ± 12.48 HU), and significantly improved SNR (31.42 ± 5.81 for DLR vs. 19.15 ± 4.48 for hybrid IR) and CNR (50.59 ± 7.62 for DLR vs. 24.08 ± 3.81 for hybrid IR). Image sharpness was significantly (*p* = 0.001) improved with DLR (0.76 ± 0.09) compared to hybrid IR (0.59 ± 0.09). Subjective image analysis revealed enhanced visualization of trabecular architecture, superior delineation and integrity of cortical bone, and improved depiction of bony cortical lesions with DLR compared to hybrid IR. **Conclusions:** Ultra-low-dose CT with DLR offers a superior approach to enhance image quality, making it a valuable tool for clinical practice.

## Simple Summary

Ankle and foot fractures are common injuries, but computed tomography exposes patients to ionizing radiation, which is a concern, particularly for those requiring repeated imaging. This study investigated whether a deep learning image reconstruction technique could improve image quality while maintaining an ultra-low radiation dose. We found that this approach substantially reduced image noise and enhanced visualization of bone structures and fracture lines compared with the conventional reconstruction method. These findings provide a basis for further prospective studies evaluating the clinical utility of advanced reconstruction techniques in ultra-low-dose CT.

## 1. Introduction

Acute ankle and foot injuries are increasingly prevalent, frequently presenting in emergency departments and accounting for a significant proportion of lower extremity trauma cases [1]. These injuries, if not promptly and accurately diagnosed, can lead to long-term physical impairments such as chronic pain, instability, joint dysfunction, and reduced mobility, as well as psychological consequences including decreased quality of life [2,3]. Therefore, early and accurate assessment is critical for initiating appropriate treatment. Conventional radiographic views, including anteroposterior, lateral, oblique, and mortise projections, are frequently used as the initial modality for diagnosing ankle and foot fractures [4]. However, fractures in these areas are often subtle or involve complex anatomy, making them prone to underestimation or misdiagnosis on plain radiographs [5,6,7]. In contrast, computed tomography (CT) provides superior sensitivity and specificity, allowing comprehensive assessment of complex fractures, dislocated joint facets, and surrounding structures, thereby serving as a critical modality for guiding appropriate management strategies, including surgical fixation or conservative treatment.

Despite these advantages, concerns about radiation exposure from CT imaging remain, particularly in younger patients who are more sensitive to ionizing radiation. To address these concerns, the use of lower tube voltage has been implemented in several studies to reduce radiation doses to levels comparable to those of conventional radiographs [8,9]. Although the ultra-low-dose (ULD) CT protocols (effective dose < 1 μSv) using low tube voltage have shown higher fracture detection rates than conventional radiography [9], studies comparing ULD CT with standard-dose CT have reported comparable diagnostic performance [10,11]. However, ULD CT protocols are associated with increased image noise and reduced image quality. This limitation persists even with the application of iterative reconstruction (IR) techniques, due to the inherent tradeoff between tube voltage reduction and image noise [10,11].

Recently, deep learning image reconstruction (DLR) has emerged as a promising solution in clinical practice. DLR utilizes deep convolutional neural networks on high-quality reference datasets to generate images with enhanced noise reduction and improved spatial resolution. Commercially available DLR methods include Advanced Intelligent Clear-IQ Engine (AiCE, Canon Medical Systems), trained on model-based iterative reconstruction data, as well as TrueFidelity (GE Healthcare) and Precise Image (Philips), both trained on FBP datasets [12,13]. DLR has superior image quality by effectively denoising and has demonstrated higher subjective analysis results when compared to traditional filtered-back projection and IR [14,15,16,17,18]. Several studies have reported that DLR significantly enhances image quality by reducing image noise, increasing the signal-to-noise ratio (SNR), and improving the contrast-to-noise ratio (CNR), even under low tube voltage settings [19,20]. However, to date, no study has specifically investigated the application of DLR in a ULD CT protocol (effective dose < 1 μSv) for patients with ankle and foot fractures. Therefore, this study aims to comprehensively evaluate the quantitative and qualitative performance of DLR in patients with ankle and foot fractures using a ULD CT protocol, compared to conventional hybrid IR.

## 2. Materials and Methods

This retrospective study was approved by the Institutional Review Board (IRB: CR325085; 23 June 2026), with a waiver of written informed consent. Patients with suspected ankle and foot fractures who underwent CT at our institution (*n* = 40), including referrals from the Department of Radiology and Orthopedic Surgery, between December 2023 and January 2025, were included. Exclusion criteria included absence of raw data (*n* = 2), age under 18 years (*n* = 4), and examinations performed with dual-energy CT (*n* = 2) (Figure 1). Fracture status was confirmed based on clinical imaging evaluation, including CT and available radiographic findings, together with the clinical diagnosis documented in the medical record. Fracture-negative examinations were not excluded.

### 2.1. Image Acquisition

All patients underwent ULD CT scan using a 320-detector-row CT (Aquilion ONE PRISM Edition, Canon Medical Systems Corporation, Otawara-si, Japan). Imaging was performed with volume scanning at a tube voltage of 80 kVp, tube current of 15 mA, display field of view of 200 mm, scan range of 160 mm, detector collimation of 160 × 0.5 mm, and rotation time of 1 s. For foot CT, the scan range extended from the hindfoot to the tips of the toes, whereas for ankle CT, the scan range extended from the distal tibial shaft to the hindfoot. The images were reconstructed using deep learning image reconstruction (DLR; Advanced Intelligent Clear-IQ Engine, AiCE, Canon Medical Systems Corporation, Otawara-si, Japan) with the bone reconstruction option with standard strength and hybrid iterative reconstruction (Hybrid IR, Adaptive Iterative Dose Reduction—3D, AIDR-3D, Canon Medical Systems Corporation, Otawara-si, Japan) with the bone reconstruction option and standard strength. Images were reconstructed with a 512 × 512 matrix with a 0.5 mm slice thickness and reconstruction interval.

### 2.2. Dose Calculation

The dose-length product (DLP, mGy·cm) and computed tomography dose index (CTDI_vol_, mGy) for the ULD CT were collected for each patient. The effective dose (ED, mSv) was estimated by multiplying the DLP by a conversion coefficient of 0.0002, as referred to in previous studies [21,22].

### 2.3. Image Analysis

The image noise, measured in Hounsfield units (HUs), was defined as the standard deviation (SD) of attenuation, with a 50 mm^2^ region of interest (ROI) placed in the subcutaneous fat. Additionally, a 40 mm^2^ ROI was placed in the distal tibia proximal to the ankle joint to measure the bone attenuation, and another 40 mm^2^ ROI was placed in the muscle at the same anatomical level. Bilateral ROIs were placed on three consecutive axial slices, and the mean attenuation value across the three slices was used for analysis. All ROI measurements were performed by a radiologist with 5 years of radiologic experience. The same anatomical locations and ROI placement procedures were applied to both hybrid IR and DLR images. The signal-to-noise ratio (SNR) for bone was calculated as the ratio of the mean bone attenuation to its corresponding standard deviation (SD). The contrast-to-noise ratio (CNR) was determined by calculating the difference in attenuation between the bone and muscle, divided by the image noise. Histogram analysis was performed at the tibial epiphysis and fibular cortex using the Medical Processing, Analysis, and Visualization (MIPAV) software version 11.0.3 (National Institutes of Health; https://mipav.cit.nih.gov/, accessed on 22 August 2026).

Image sharpness was quantitatively assessed using the perceptual sharpness index (PSI), a no-reference metric grounded in local edge gradient analysis developed by Feichtenhofer et al. [23]. The analysis was performed using an automated in-house program developed in Python 3.11.9 with NumPy 1.26.4, based on the publicly available MATLAB R2026a implementation (The MathWorks, Inc., Natick, MA, USA) (https://github.com/feichtenhofer/PSI, accessed on 22 August 2026). This algorithm evaluates edge responses within a localized region of the image and characterizes their distribution to perceptual sharpness. Before PSI calculation, each image was standardized to zero mean and unit variance using $I_{norm}=(I-\;\mu_{1}/\sigma_{1})$. Sharpness evaluation was conducted for every sagittal slice in each original CT dataset, encompassing the tibia, calcaneus, talus, and navicular bones, without image cropping or manual region-of-interest placement. The algorithm was applied using its default parameters: percentile, 22%; block size, 32 pixels; and block width threshold, 2. No image cropping or manual region-of-interest placement was performed. The same processing procedure was applied to both reconstruction methods. Higher metric values correspond to sharper images, reflecting more distinct structural boundaries and contrast transitions.

The subjective image analysis was independently conducted by two readers: a musculoskeletal radiologist with 8 years of experience and an orthopedic surgeon with 12 years of experience. All images were anonymized and presented to the observers without information regarding the reconstruction technique. Subjective image-quality assessment was performed once for each patient on each reconstructed image set. The images were evaluated in a randomized order to minimize potential recognition of the reconstruction method and reader bias. Both observers were blinded to the reconstruction technique and were allowed to freely adjust the window level and width. Each observer rated the images using a 5-point scale as follows: 5—excellent image quality with very low image noise, no artifact, excellent depiction of bony cortical lesions, excellent trabecular architecture visualization, and excellent cortical bone delineation/integrity; 4—good image quality with low image noise, almost no artifact, good depiction of bony cortical lesions, good trabecular architecture visualization, and good cortical bone delineation/integrity; 3—moderate image quality with moderate image noise, moderate artifact, adequate depiction of bony cortical lesions, moderate trabecular architecture visualization, and moderate cortical bone delineation/integrity; 2—poor image quality with severe image noise, severe artifact, poor depiction of bony cortical lesions, poor trabecular architecture visualization, and poor cortical bone delineation/integrity; 1—very poor image quality with unacceptable image noise, very severe artifact, very poor depiction of bony cortical lesions, very poor trabecular architecture visualization, and very poor cortical bone delineation/integrity [10,24]. Each item was rated separately using a 5-point ordinal scale, with higher scores indicating better image quality.

### 2.4. Statistical Analysis

The Kolmogorov–Smirnov and Shapiro–Wilk tests were used to assess the normality of the data. Continuous variables were reported as mean and SD or median and interquartile range (IQR), as appropriate. Quantitative image-quality parameters were compared between hybrid IR and DLR using the paired *t*-test. Subjective image-quality scores were compared using the Wilcoxon signed-rank test. Interobserver agreement for the subjective image-quality assessments was assessed across the overall set of subjective scoring items using a two-way mixed-effects, single-measure, absolute-agreement intraclass correlation coefficient (ICC). The overall ICC was reported with its 95% confidence interval. ICC values were categorized as follows: <0.21 (poor), 0.21–0.40 (fair), 0.41–0.60 (moderate), 0.61–0.80 (strong), and >0.80 (near-complete agreement). A *p*-value of <0.05 was considered statistically significant. Statistical analyses were performed using the Statistical Package for the Social Sciences version 25.0 (IBM, Armonk, NY, USA).

## 3. Results

A total of 32 patients (mean age, 54 ± 16 years; range, 18–79 years; 18 men) were included in this study. Fourteen patients underwent observation, while 18 underwent surgery, including internal fixation (Table 1). The mean CTDI was 0.11 ± 0.02 mGy, while the mean DLP was 4.30 ± 0.57 mGy·cm for foot and ankle CT. The effective dose was 0.86 ± 0.11 μSv.

Table 2 summarizes the image noise, CT attenuation, SNR, and CNR for the two image reconstruction methods. The image noise in HU was significantly (*p* = 0.001) reduced with DLR (47.33 ± 6.60) compared to hybrid IR (87.65 ± 12.48). There was no significant (*p* = 0.58) difference in CT attenuation of the muscle between hybrid IR (61.04 ± 12.53) and DLR (60.40 ± 8.29), while the measured CT attenuation of the bone was significantly higher with DLR than with hybrid IR (2409 ± 112.30 vs. 2131 ± 160.30, respectively; *p* = 0.001).

The mean SNR was significantly (*p* = 0.001) higher for DLR (31.42 ± 5.81) compared to hybrid IR (19.15 ± 4.48), even in the ULD protocol. Similarly, the CNR was significantly (*p* = 0.001) improved for ULD CT with DLR (50.59 ± 7.62) compared with ULD CT with hybrid IR (24.08 ± 3.81) (Table 2).

In a representative patient, the histogram analysis demonstrated that DLR yielded a higher peak CT attenuation value with a sharper peak compared to hybrid IR in the tibial epiphysis. This resulted in a higher peak around 500 HU for DLR, which enhanced the visualization of trabecular architecture by improving the clarity and definition of fine structural detail. On the other hand, hybrid IR exhibited a broader and smaller peak. Regarding the cortical bone, DLR exhibited superior noise suppression, as indicated by a more uniform distribution of CT attenuation values. DLR had a smaller standard deviation (207.20 vs. 283.10), indicating less variability in attenuation compared to hybrid IR (Figure 2). Image sharpness was significantly (*p* = 0.001) improved with DLR (0.76 ± 0.09) compared to hybrid IR (0.59 ± 0.09) (Table 2).

DLR received higher overall image-quality scores than hybrid IR (4.0 (4.0–4.0) vs. 2.0 (2.0–3.0), *p* = 0.001). For image-noise assessment, all scores were identical within each reconstruction group (3.0 (3.0–3.0) for DLR vs. 2.0 (2.0–2.0) for hybrid IR). In terms of anatomical detail, DLR demonstrated significantly enhanced visualization of trabecular architecture (3.0 (3.0–3.0) vs. 2.0 (2.0–2.0)), superior cortical bone delineation and integrity (4.0 (4.0–4.0) vs. 3.0 (2.0–3.0)), and improved depiction of bony cortical lesions (4.0 (4.0–4.0) vs. 3.0 (3.0–3.8)), all with statistically significant differences (*p* = 0.001). There was no significant difference in artifact scores between DLR and hybrid IR (*p* = 0.50), suggesting comparable artifact suppression capabilities. Interobserver agreement was excellent, as indicated by an ICC of 0.965 (95% confidence interval: 0.958–0.972) (Table 3). Representative cases are shown in Figure 3 and Figure 4. The bone fracture lines and fragments were more clearly visualized with DLR compared to hybrid IR.

## 4. Discussion

The results of this study demonstrated that ULD CT with DLR improves both qualitative and quantitative image quality in patients with ankle and foot fractures compared to hybrid IR.

To reduce undesirable radiation-related effects, several studies have recommended the use of a combination of low tube voltage scanning and iterative reconstruction to achieve ULD imaging [9,10,11]. The effective radiation dose in this study was approximately 0.86 ± 0.11 μSv, which is approximately 40 times lower than that of the standard-dose CT [10], and lower than that reported in a previous ULD CT study [9]. The ULD CT protocol employed in this study demonstrated radiation exposure levels comparable to conventional radiography [25]. Furthermore, the use of ULD CT is particularly beneficial for patients with ankle and foot fractures who may necessitate serial imaging for monitoring fracture healing or preoperative planning, as it significantly reduces cumulative radiation dose. Although the absolute radiation exposure from the present protocol was extremely low, minimizing unnecessary radiation exposure remains important because epidemiologic studies have demonstrated potential long-term health risks associated with ionizing radiation exposure from CT, particularly in more radiosensitive populations [26,27]. The higher diagnostic performance of ULD CT for fracture evaluation has been reported in studies [9,10,11] compared with general radiography. Alagic et al. found that ULD CT identified 15.9% more fracture-related findings and 4.3% more non-acute-fracture-related findings in the extremities compared with conventional radiography [9]. Also, ULD CT led to treatment modifications, upgrading management from functional treatment to cast immobilization in 18 out of 207 patients, where these findings were not detected on conventional radiograph. However, Xiao et al. reported comparable diagnostic accuracy, sensitivity, and specificity between ULD CT and standard-dose CT [10]. Notably, the ULD CT protocol exhibited significantly higher image noise (162.49 ± 10.11) compared to the standard dose (41.39 ± 10.03). Although previous ULD CT protocols significantly reduced radiation doses and improved treatment options, they utilized hybrid IR, which often resulted in increased image noise and reduced overall image quality. In contrast, the present study applied DLR at a comparable radiation dose, with the goal of enhancing image quality. Compared to hybrid IR, DLR reduced image noise by approximately 1.8-fold while improving the SNR and CNR by approximately 1.6-fold and 2.1-fold, respectively. Furthermore, histogram analysis demonstrated that DLR improved the visualization of trabecular architecture by enhancing the clarity and definition of fine structural detail. DLR also demonstrated significantly higher bone HU values than hybrid IR; however, this difference may be related to reconstruction-dependent changes in edge characteristics. Therefore, quantitative CT attenuation measurements should be interpreted with caution when comparing images reconstructed using different techniques, as reconstruction-dependent changes may influence measured HU values. Subjective evaluations by both observers consistently favored DLR over hybrid IR in terms of overall image quality, depiction of trabecular and cortical bone architecture and visualization of bony cortical lesions. These findings suggest that DLR enhances image quality relative to the widely used hybrid IR.

The use of ULD CT with DLR may offer several advantages in routine clinical practice. The visualization of intra-articular fractures of the foot and ankle is often hindered or underestimated on conventional radiographs due to their lower resolution and restricted projection angles. ULD CT with DLR may facilitate fracture detection by improving the visualization of fracture lines and small fracture fragments; however, this potential benefit remains to be established in future prospective studies. This is particularly important because delayed or missed diagnosis of complex fractures can adversely affect patient outcomes. Notably, the no-reference perceptual sharpness metric was significantly higher with DLR compared to hybrid IR, highlighting its superior ability to improve sharpness.

This study has several limitations. First, the retrospective design and small sample size limit the generalizability of our findings. Second, the present study evaluated image quality and subjective visualization of fracture-related osseous structures rather than fracture detection or diagnostic accuracy. Because fracture-negative examinations were not included, sensitivity, specificity, accuracy, and fracture detection rates could not be assessed. Third, the no-reference perceptual sharpness metric used in this study was originally developed as a general image-processing measure and has not been specifically validated for fracture detection. Additionally, a fixed tube voltage of 80 kVp and tube current of 15 mA were used for all patients without automatic tube current modulation. Nevertheless, our findings demonstrate that ULD CT reconstructed with DLR provides improved quantitative and qualitative image quality compared with hybrid IR in patients with ankle and foot fractures. Furthermore, all results were limited to a single vendor and a single image reconstruction method. In this study, ULD CT combined with DLR demonstrated improved quantitative and qualitative image quality in the foot and ankle compared with hybrid IR. Further prospective studies are warranted to determine whether these image-quality improvements translate into improved diagnostic performance.

## 5. Conclusions

ULD CT with DLR provides improved quantitative and qualitative image quality compared with hybrid IR in patients with ankle and foot fractures.

## Figures and Tables

**Figure 1:**
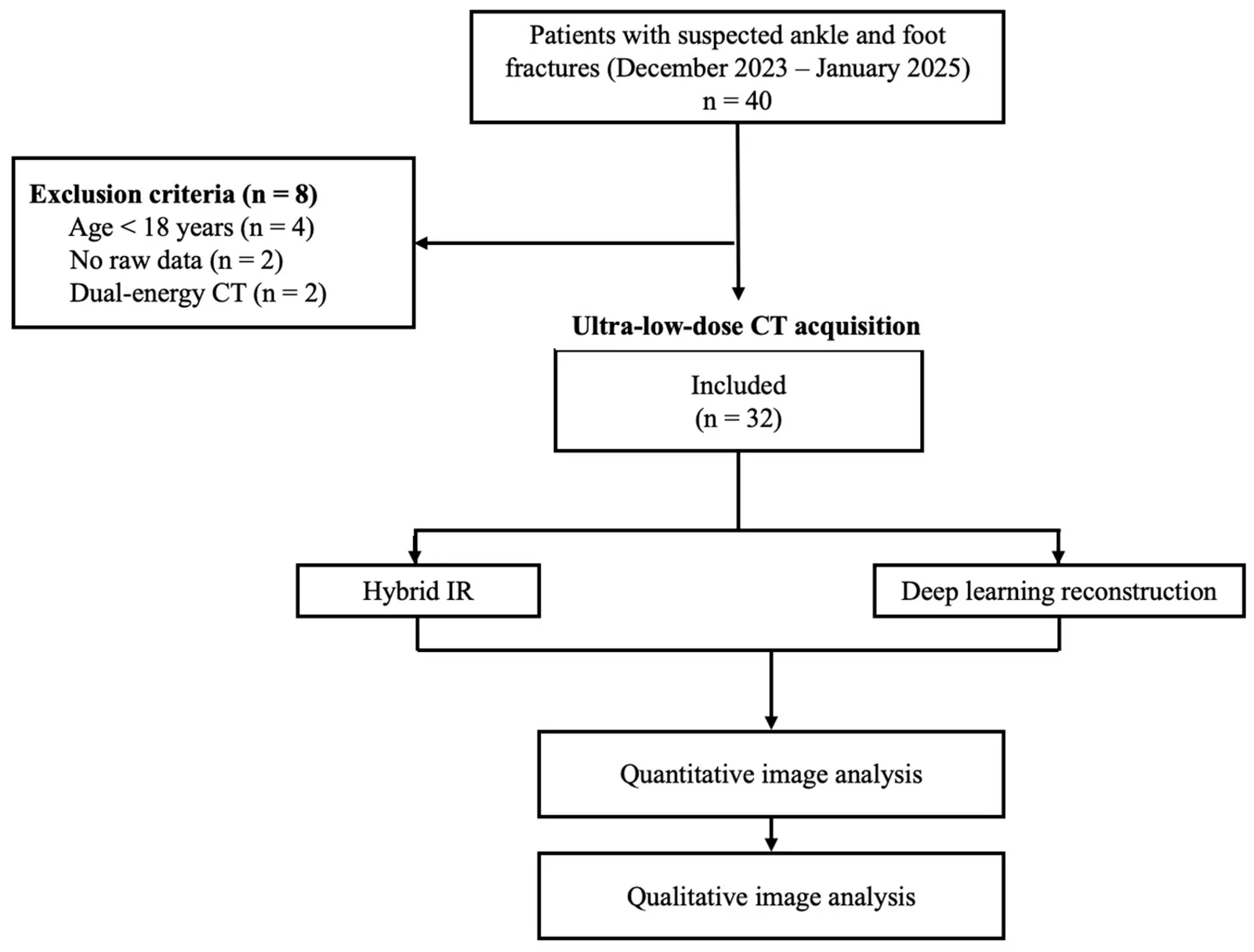
Study flowchart of patient selection. Patients with suspected ankle and foot fractures who underwent CT examinations between December 2023 and January 2025 were retrospectively screened. Among 40 eligible patients with suspected ankle and foot fractures, 8 patients were excluded due to age younger than 18 years (n = 4), absence of raw data (n = 2), and dual-energy CT acquisition (n = 2). The final study population consisted of 32 patients.

**Figure 2:**
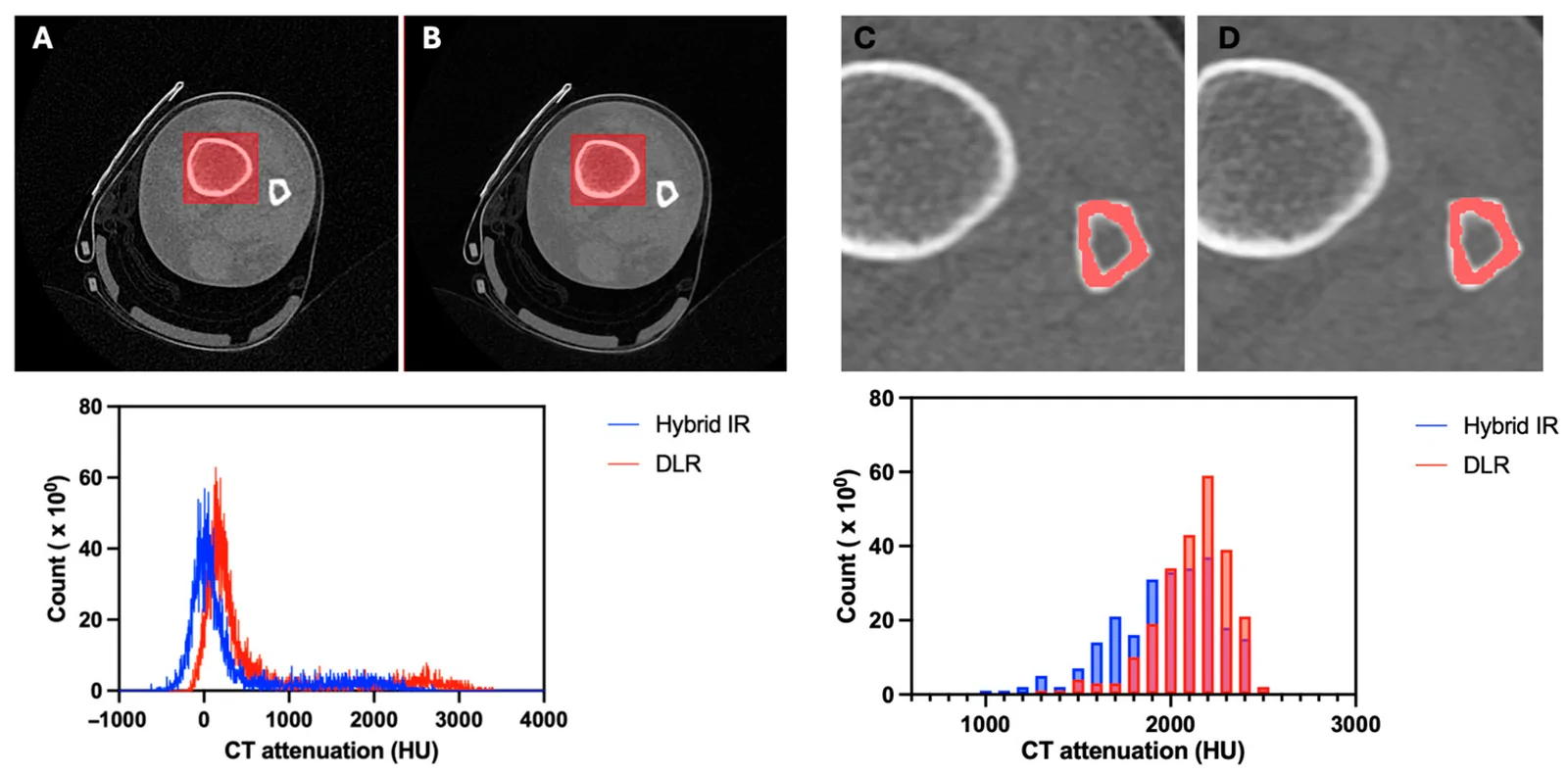
Histogram analysis between hybrid iterative reconstruction (IR) and deep learning image reconstruction (DLR). (**A**,**B**) Tibial epiphysis images and histograms. (**C**,**D**) Cortical bone images and histograms. The (**left** panel) shows that DLR (red line) produced a more prominent and narrower peak centered at approximately 500 HU than hybrid IR (blue line) for the tibial epiphysis (**top right** panel), corresponding to improved visualization of the trabecular architecture. In the cortical bone (**bottom right** panel), DLR demonstrated better noise suppression, with more uniform HU distribution and a smaller standard deviation (207.20 vs. 283.10) than hybrid IR. The red circles and boxes indicate the masks used for histogram analysis.

**Figure 3:**
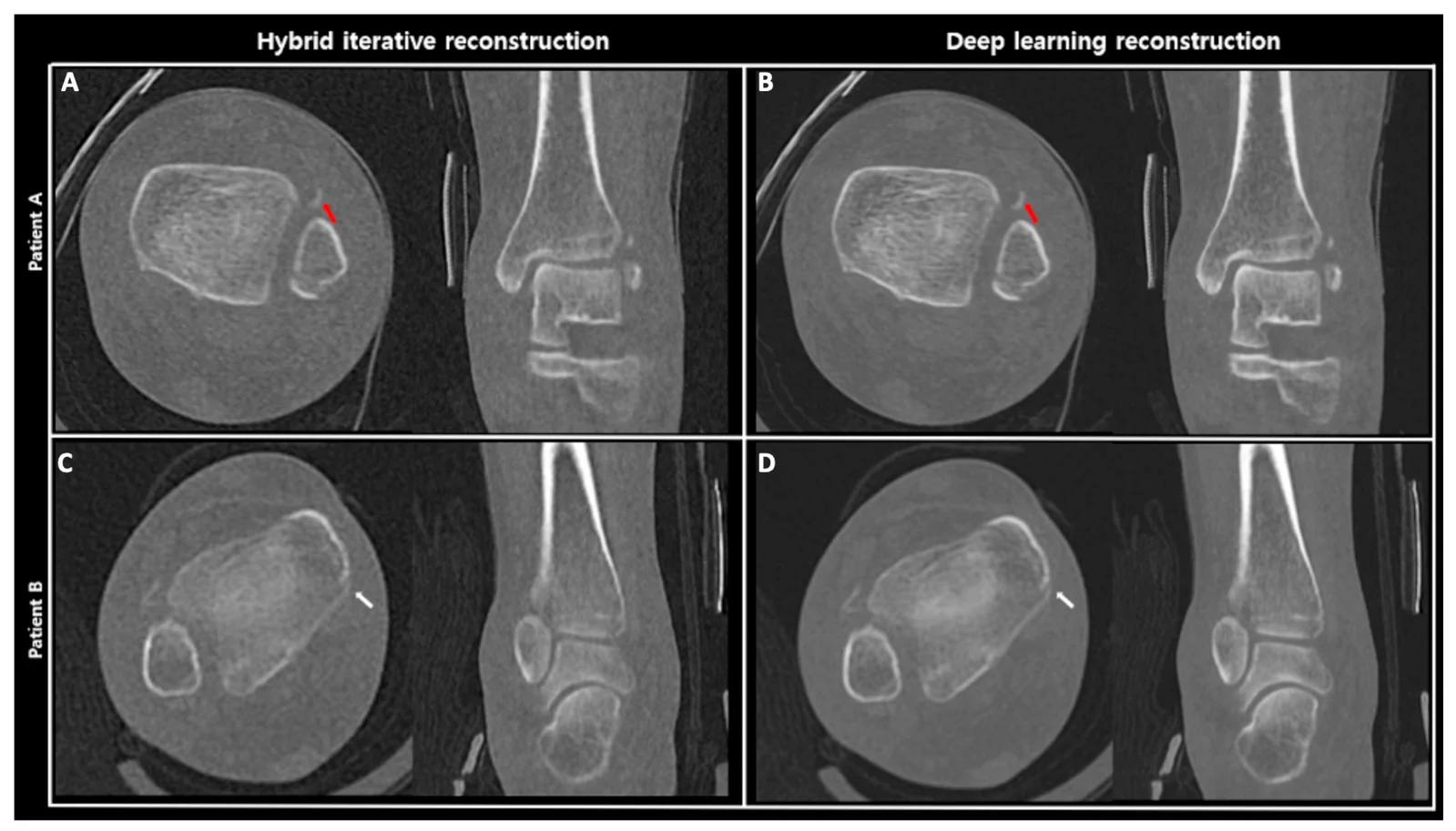
Comparison between hybrid iterative reconstruction and deep learning reconstruction (right) in two patients (A and B) undergoing ULD CT. (**A**,**B**) Patient A; (**C**,**D**) Patient B. Axial and coronal images demonstrate improved sharpness of fracture fragments (red arrows) and fracture lines (white arrows) with clearer cortical details and lower image noise using deep learning reconstruction. In contrast, images reconstructed with hybrid iterative reconstruction show more irregular contours and reduced sharpness in both patients A and B.

**Figure 4:**
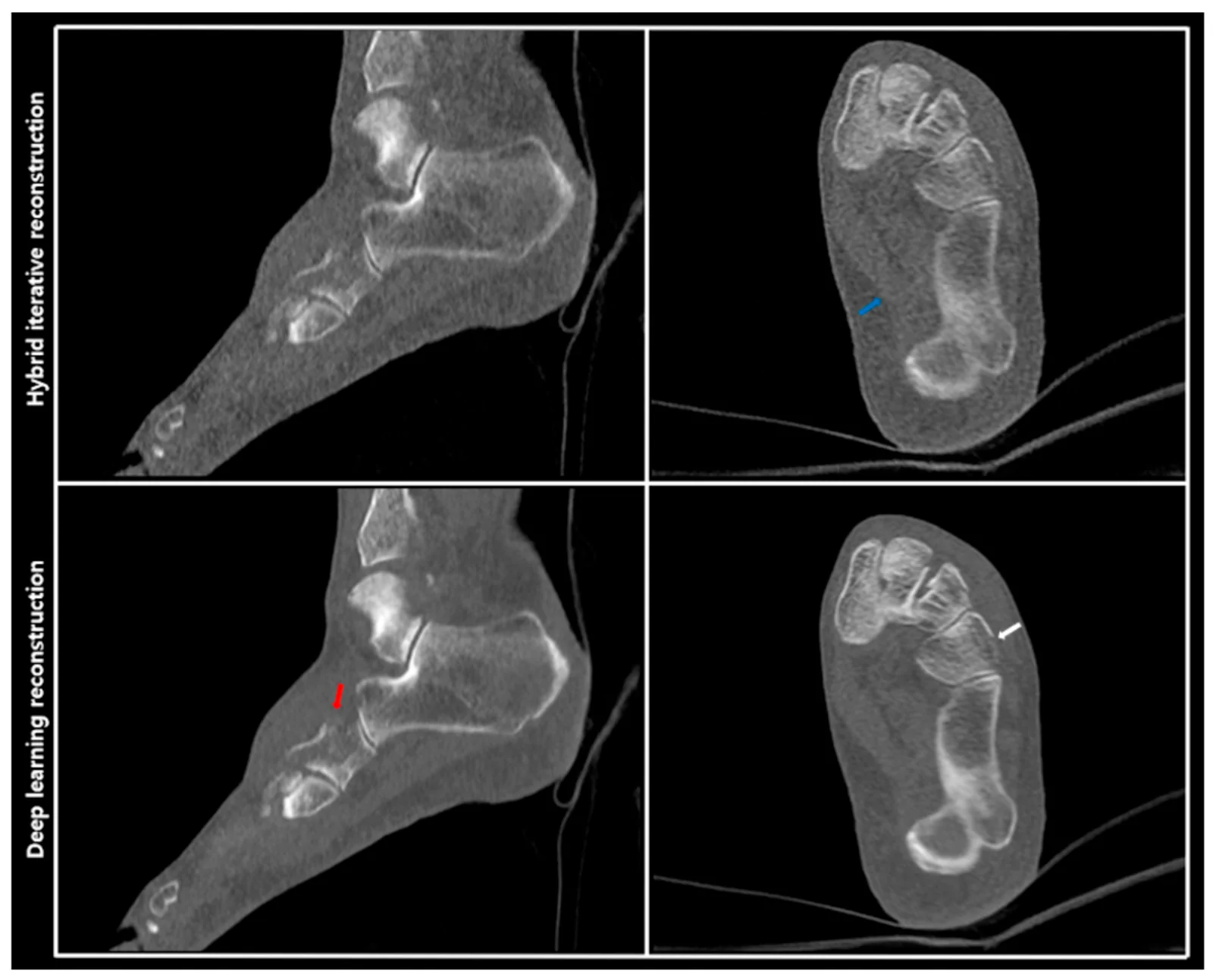
Representative case of a patient with a bone fracture. Images reconstructed with hybrid iterative reconstruction (IR) and deep learning reconstruction (DLR) correspond to the same anatomical level. The fracture line (white arrow) is visible on both reconstructions but is more clearly delineated, while the small fracture fragment (red arrow) is more conspicuously visualized on DLR. Hybrid IR demonstrates higher image noise and less homogeneous depiction of the surrounding fat and muscle tissues (blue arrow) than DLR.

**Table 1:** Patient characteristics.

Characteristics	Number of Patients (n = 32)
Age (years)	54 ± 16
Gender	
Male, n (%)	18 (56%)
Female, n (%)	14 (44%)
Total number of fracture sites	80
Total fracture sites	
Ankle	20
Foot	60

Data are reported as mean ± standard deviation.

**Table 2:** The results of image noise, CT attenuation, SNR, CNR, and image sharpness.

	Hybrid IR	DLR	*p* Value
Image noise (HU)	87.65 ± 12.48	47.33 ± 6.60	0.001
CT attenuation_(bone)_ (HU)	2131.00 ± 160.30	2409.00 ± 112.30	0.001
CT attenuation_(muscle)_ (HU)	61.04 ± 12.53	60.40 ± 8.29	0.58
SNR	19.15 ± 4.48	31.42 ± 5.81	0.001
CNR	24.08 ± 3.81	50.59 ± 7.62	0.001
Image sharpness	0.59 ± 0.09	0.76 ± 0.09	0.001

Data are reported as mean ± standard deviation. HU, Hounsfield Unit; SNR, signal-to-noise ratio; CNR, contrast-to-noise ratio; IR, iterative reconstruction; DLR, deep learning image reconstruction.

**Table 3:** The results of subjective image analysis.

	Overall Image Quality	Image Noise	Artifact	Trabecular Architecture	Cortical Bone Delineation/ Integrity	Depiction of the Bony Cortical Lesion
Observer 1						
Hybrid IR	2.0 (2.0–3.0)	2.0 (2.0–2.0)	4.0 (4.0–4.0)	2.0 (2.0–2.0)	3.0 (3.0–3.0)	3.0 (3.0–4.0)
DLR	4.0 (4.0–4.0)	3.0 (3.0–3.0)	4.0 (4.0–4.0)	3.0 (3.0–3.0)	4.0 (4.0–4.0)	4.0 (4.0–4.0)
*p* value	0.001	N/A	0.50	0.001	0.001	0.001
Observer 2						
Hybrid IR	2.0 (2.0–2.3)	2.0 (2.0–2.0)	4.0 (4.0–4.0)	2.0 (2.0–2.0)	3.0 (2.0–3.0)	3.0 (3.0–3.8)
DLR	4.0 (4.0–4.0)	3.0 (3.0–3.0)	4.0 (4.0–4.0)	3.0 (3.0–3.0)	4.0 (4.0–4.0)	4.0 (4.0–4.0)
*p* value	0.001	N/A	N/A	0.001	0.001	0.001
ICC	0.965 (0.958–0.972, *p* = 0.001)					

Data are reported as median (interquartile range). IR, iterative reconstruction; DLR, deep learning image reconstruction; ICC, intraclass correlation coefficient.

## Data Availability

The data presented in this study are not publicly available due to privacy and ethical restrictions related to patient data but are available from the corresponding author upon reasonable request.

## Abbreviations

The following abbreviations are used in this manuscript:

CNR	Contrast-to-Noise Ratio
CT	Computed Tomography
CTDI	Computed Tomography Dose Index
DLP	Dose-Length Product
DLR	Deep Learning Reconstruction
ED	Effective Dose
HU	Hounsfield Unit
ICC	Intraclass Correlation Coefficient
IR	Iterative Reconstruction
SNR	Signal-to-Noise Ratio
ULD	Ultra-Low-Dose
